# Editor's Letter-Box

**Published:** 1896-10-24

**Authors:** 


### EDITOR'S LETTER-BOX.

[Our Correspondents are reminded that prolixity is a great bar to publication, and that brevity of style and conciseness of statement greatly facilitate early insertion.]

Dr. Braidwood, whose pamphlet on this subject we noticed some time ago, writes, urging that such ships should be an integral part of the fleet in naval war; that a certain number of such ships should be built by the Government, to be subject to the same rules and regulations as are at present in force in vessels of the naval service. He considers that hospital ships built after his plan could be used as ambulance ships, as isolation hospitals, stationed off unhealthy coasts, and as convalescent hospitals, moored near large towns. In war, however, they would accompany the fleet. As to such details as the ventilation, heating, and lighting of various cabins and wards, the omission of which we noticed, he says he has purposely not arranged for nor discussed them, as such things belong to the department of the naval architect.

